# Crystallin Alpha-B Overexpression as a Possible Marker of Reactive Astrogliosis in Human Cerebral Contusions

**DOI:** 10.3389/fncel.2022.838551

**Published:** 2022-03-14

**Authors:** Lina Vanessa Becerra-Hernández, Martha Isabel Escobar-Betancourt, Hernán José Pimienta-Jiménez, Efraín Buriticá

**Affiliations:** Centro de Estudios Cerebrales, Facultad de Salud, Universidad del Valle, Cali, Colombia

**Keywords:** crystallin alpha B, CRYAB, αB-crystallin, reactive gliosis (astrogliosis), brain contusion, severe traumatic brain injury (TBI), glial fibrillary acidic protein (GFAP), human cerebral cortex

## Abstract

The pathophysiology of traumatic brain injury (TBI) has not yet been fully elucidated. Crystallin alpha-B (CRYAB) is a molecular chaperone that apparently tries to stabilize the rapid thickening of the intermediate filaments of glial fibrillary acidic protein (GFAP) during the process of reactive astrogliosis in response to TBI. Previous analyses of the gene expression profile in human brain contusion tissue showed us an exacerbated CRYAB overexpression. Here, we used 3, 3’-diaminobenzidine (DAB) immunohistochemistry and immunofluorescence to verify CRYAB overexpression and to describe its expression and distribution in samples of contused cortical tissue derived from emergency decompressive surgery after severe TBI. The histological expression of CRYAB was mainly seen in subcortical white matter astrocytes of injured tissue. Most of the cells that overexpressed GFAP in the analyzed tissue also overexpressed CRYAB, a finding corroborated by the co-localization of the two markers. The only difference was the presence of a few pyramidal neurons that expressed CRYAB in layer V of the cerebral cortex. The selective vulnerability of layer V of the cerebral cortex during TBI could explain the expression of CRYAB in neurons of this cortical layer. Our results indicate a parallel behavior in the cellular expression of CRYAB and GFAP during the subacute response to TBI. These results lead us to postulate CRYAB as a possible marker of reactive astrogliosis in contused cortical tissue.

## Introduction

Traumatic brain injury (TBI) is a phenomenon that involves a direct injury caused at the time of the event (primary damage), as well as injuries caused by secondary damage in the aftermath of the event, which are closely related to trauma sequels and patient outcome. Various cellular and molecular events involved in the establishment and progress of secondary damage due to trauma have been described (Maas, 2016; O’Leary and Nichol, 2018). The phenomenon of reactive astrogliosis is one of these events, during which changes in glial cells in response to injury can be seen. Astrocytes are a type of glial cell. They are abundant in the nervous system, with functions related to the maintenance of tissue homeostasis, and the isolation of synaptic clefts. Reactive astrocytes are morphologically characterized by increased cell size, compared with astroglial cells at rest, as well as increased production of cytoskeletal intermediate filaments. Consequently, Glial Fibrillary Acidic Protein (GFAP) staining has been widely used to identify reactive glia in a variety of central nervous system diseases including TBI (Pekny and Pekna, 2014). Although an increase in intermediate filaments has been evident in the phenomenon of reactive astrogliosis, it has been noted that all functional changes experienced by astroglial cells in response to injury are considered part of the phenomenon of astrogliosis, whose characteristics may vary according to the injury (Wang and Bordey, 2008; Kimelberg and Nedergaard, 2010). Cerebral edema, the increase in water in brain tissue, is another secondary phenomenon of TBI in which astrocytes are involved. Cerebral edema following central nervous system injury has classically been categorized as either “vasogenic” or “cytotoxic” although these processes have been increasingly recognized as possibly interrelated (Simard et al., 2007; Salman et al., 2022). Cytotoxic edema is generated by the failure of ion pumps and/or by the activation of selected ion channels, with the consequent loss of homeostatic ion gradients. This causes cellular swelling, in which water moves from the interstitial space into the interior of the cell (Salman et al., 2022). Vasogenic edema, on the other hand, refers to the change in the disposition of fluids in the blood vessels, secondary to the mechanical compromise of the blood–brain barrier, added to the associated inflammatory phenomena (Simard et al., 2007).

HSPB5 or crystallin alpha-B (CRYAB) is a protein that belongs to the family of small heat shock proteins (sHSPs). It is widely distributed in the nervous system and is found mainly in astrocytes, where it takes on multiple roles in cell survival pathways and the maintenance of homeostasis (Tikhomirova et al., 2017). This protein is highly protective against oxidative stress (Lassen et al., 2008; Malin et al., 2016; Yu et al., 2016) and promotes cell survival and proliferation by modulating PI3K/Akt/mammalian target of rapamycin (mTOR) pathways (Wang et al., 2012; Zhu et al., 2015). In addition, it confers protection against apoptosis by inhibiting the proteolytic activation of caspase 3 and binding to pro-apoptotic members of the Bcl-2 family (Kamradt et al., 2001; Mao et al., 2004; Shin et al., 2009). The anti-apoptotic properties of CRYAB have been associated with the resistance to apoptosis of tumor cells (Kamradt et al., 2005; Voduc et al., 2015; Malin et al., 2016), and its expression has been identified as a predictor of poor survival in colorectal and breast cancer (Moyano et al., 2006; Shi et al., 2014).

In some studies, astrocytes have been the viable therapeutic target in pathologies of the nervous system (Liu et al., 2015; Smethurst et al., 2020), and it has been demonstrated that CRYAB has the ability to dissolve toxic protein aggregates such as amyloid beta in Alzheimer’s disease, alpha synuclein in the Parkinson’s disease, and GFAP in Alexander’s disease (Rekas et al., 2004; Narayanan et al., 2006; Hagemann et al., 2009; Tang et al., 2010; Cox et al., 2014). A wide variety of tauopathies that include glial diseases, such as corticobasal degeneration, progressive supranuclear palsy, and frontotemporal dementia with parkinsonism linked to chromosome 17, feature CRYAB and Tau inclusions in astrocytes (Dabir et al., 2004; López-González et al., 2014). It has also been recognized as a marker of epileptic foci in children with increased expression of glial cells (Sarnat and Flores-Sarnat, 2009). An increase in the expression of CRYAB in animal models of ischemia (Piao et al., 2005; Li et al., 2012; Bartelt-Kirbach et al., 2017) has been associated with the survival of ganglion cells in the retina after axotomy of the optic nerve in mice (Munemasa et al., 2009). It has also been demonstrated that administration of recombinant human CRYAB in the first week postlesion in murine models of spinal cord contusion injury improves motor skills and reduces secondary tissue damage (Klopstein et al., 2012) and that CRYAB regulates remyelination after peripheral nerve injury (Lim et al., 2017).

Crystallin alpha-B has been linked to the stabilization of the GFAP protein in glial cells of the central nervous system (Wang and Spector, 1996; Koyama and Goldman, 1999; Xi et al., 2006). The coexisting overexpression of GFAP and CRYAB has been shown for Rosenthal fibers in Alexander’s disease, chronic glial scars, and low- and high-grade fibrillar astrocytomas (Der et al., 2006; Hagemann et al., 2006; Goplen et al., 2010; Sosunov et al., 2017).

The expression of CRYAB in TBI has not been previously evaluated. This study demonstrates the upregulation of CRYAB in human TBI and its relationship with GFAP protein expression during the phenomenon of reactive astrogliosis secondary to trauma. Data obtained previously by our research group indicated an increase in the expression of the CRYAB gene, from which the protein CRYAB is encoded. These data were obtained using complementary DNA (cDNA) microarrays with contused brain tissue derived from adult human patients who had suffered TBI to the frontal or temporal lobe and underwent decompressive surgery. Strict selection criteria were used to yield a differential expression profile of 182 genes that were common to both lobes. The overexpression of CRYAB was validated by real-time PCR (see Supplementary Figure 1).

## Materials and Methods

### Samples

Samples were collected from 10 subjects who suffered from TBI and underwent decompressive craniectomy, with the removal of the contusion at the Neurosurgical Unit of the Hospital Universitario del Valle (Table 1). The tissue corresponded to the contused cortex from the frontal or temporal lobes. The procedures were approved by the Human Ethics Committee of the Faculty of Health, Universidad del Valle, and Hospital Universitario del Valle, in accordance with the Helsinki criteria.

**TABLE 1:** Demographic data of the samples with traumatic brain injury (TBI).

Subject	Age^†^	Sex^‡^	Post-TBI time^§^	Lobule^¶^	Hemisphere*
T1	71	M	2	T	R
T2	22	M	3	F	R
T3	38	M	2	F	L
T4	35	M	3	F	L
T5	53	M	1	T	R
T6	48	M	4	F	L
T7	4	F	2	T	R
T8	45	M	3	T	R
T9	62	M	3	T	L
T10	24	M	2	T	R

^†^In years; ^‡^M, Male; F, Female; ^§^In days; ^¶^F, frontal; T, temporal; *R, right; L, left.

Control tissue samples were obtained from the cerebral cortex of 3 postmortem subjects; they were provided by the Institute of Legal Medicine and Forensic Sciences of Cali and approved by the Human Ethics Committee of the Faculty of Health, Universidad del Valle. The samples came from postmortem male subjects whose cause of death did not compromise the brain; forensic examination did not show macroscopic signs of brain degeneration (Table 2).

**TABLE 2:** Demographic data of the control postmortem samples.

Subject	Age^†^	Sex^‡^	Postmortem interval^§^	Lobe^¶^	Hemisphere*	Type of injury**
C1	45	M	8	F and T	L and R	IF
C2	18	M	12	F and T	L and R	CA
C3	33	M	15	F	L and R	ISW

^†^In years; ^‡^M, Male; F, Female; ^§^ In hours; ^¶^F, frontal; T, temporal; *R, right; L, left; **IF, Injury by firearm; CA, Car accident; ISW, injury by a sharp weapon.

### 3, 3′-Diaminobenzidine Immunohistochemistry

All the brain tissue samples were immersed in 9% saline for 5 min and then fixed with 2% paraformaldehyde in phosphate-buffered saline (PBS) for 7–10 days. After immersion in 10, 20, and 30% sucrose (24 h each), the samples were frozen using 2-metylbutane (Product No. 59070, Sigma-Aldrich, Merck) and stored at −75°C until processing. In total, 30 μm thick sections of tissue were obtained using a Leica Jung Frigocut 2800N cryostat microtome, and stored in antifreeze solution (50% PBS, 30% ethylene glycol, and 20% glycerol) prior to selecting consecutive tissue sections to be processed immunohistochemically by free floating with each of the primary antibodies of interest. Sections were immersed in a solution of 30% methanol (Product No. 179337, Sigma-Aldrich, Merck) and 0.3% hydrogen peroxide (Product No. 31642, HoneyWell-Fluka) for 20 min. The adherence to non-specific antigens was blocked with normal goat serum for CRYAB and normal horse serum for NeuN and GFAP (Vectastain Elite ABC-HRP kits: products No. PK-6101 rabbit and PK-6102 mouse, respectively; Vector Labs), for 40 min. Tissue was incubated overnight in 1:100 diluted primary antibody Anti-phospho-α-B Crystallin Serine 59 produced in rabbit (Product No. C7990, Sigma-Aldrich, Merck). 1:400 diluted primary monoclonal anti-GFAP antibody (G-A-5, mouse; Product No. G3893, Sigma-Aldrich, Merck) was used for GFAP labeling and 1:2,500 diluted mouse antineuronal nuclei (NeuN) monoclonal antibody (Clone A60, Product No. MAB377, Millipore-Merck) was used for NeuN. The tissue was subsequently incubated for 40 min in 1:200 diluted biotinylated secondary antibody and Avidin-HRP solution (Vectastain Elite ABC-HRP kits: products No. PK-6101 rabbit and PK-6102 mouse; Vector Labs). A solution containing 4% diaminobenzidine, hydrogen peroxide, and 2% nickel in PBS (Peroxidase Substrate Kit; 3, 3’-diaminobenzidine (DAB) SK-4100, Vector Labs) was then added for 7 min. Three 5 min washes using PBS were undertaken between each reagent incubation. The tissue was mounted on slides that were dried overnight and then covered with mounting medium (Permount Mounting Media, Product No. SP15-500, Fisher Chemical) and coverslips.

### Immunofluorescence

Six tissue sections from three contused subjects were chosen for immunofluorescent free-floating double-labeling, due to the scarcity of available tissue. These sections were immersed in 50 mM ammonium chloride for 30 min to increase the exposure of epitopes. Adhesion of non-specific antigens was blocked for 1 h with bovine serum albumin (BSA) (Product No. 0332-100G, Amresco). Overnight incubation was performed using the primary antibodies, 1:400 diluted anti-GFAP, and 1:100 diluted anti-CRYAB (the same antibodies used for DAB-immunohistochemistry). Both antibodies were incubated simultaneously to obtain double-labeling. Each well was incubated the next day for 2 h with secondary antibodies 1:2,000 diluted goat anti-mouse Alexa Fluor 488 for GFAP (goat antimouse IgG1 cross-adsorbed secondary antibody, molecular probes/Invitrogen, product No. A21121) and 1:2,000 diluted goat antirabbit Alexa Fluor 594 for CRYAB (goat antirabbit IgG secondary antibody, Molecular Probes/Invitrogen, product No. A11012). After the incubation of each reagent, the tissue sections were washed three times for five min with PBS, except after the fluorescent secondary antibodies, when tissue sections were washed eight times for five min with PBS, followed by one wash with distilled water for 2 min. These procedures were conducted in a dark room. Mounted tissues were dried overnight, then covered with mounting medium (Vectashield Hard set, product No. H-1400, Vector Labs). Brain tissue sections from all the control and TBI subjects were subjected to the same standard laboratory conditions.

### Image Records and Cell Counts

DAB-immunohistochemistry microphotographs were obtained with a digital camera (AxioCam HRC, Zeiss) coupled to a light microscope (Axio Scope A1, Zeiss). For cell counting, sequential photographs were taken with a 10X/0.25 objective lens, to obtained images of sectors from the pia to the deep subcortical white matter for all the three markers. These sections were then fused using Cannon Utilities Photo Stitch. A cortical column was obtained and placed on a two-dimensional 200 μm × 300 μm grid for counting (SigmaScan Pro program 5, SPSS Science, 2000). Quantitative data were obtained only for the GFAP and CRYAB markers, as DAB-immunoreactivity (IR) for NeuN was only used for the general assessment of the cerebral cortex and to identify the limits between cortical layers, given that these limits were not identifiable using the other two markers. Images of counts in homologous sectors of consecutive sections stained for GFAP and CRYAB were obtained. Four sectors were chosen to count positive cells in every section: (a) supragranular or supragranular layers (SUPRA) (between layers II and III), (b) infragranular or infragranular layers (INFRA) (specifically in layer V), (c) transition between layer VI and subcortical white matter or TRANS, and (d) deep white matter or DWM. In total, we obtained DAB-immunopositive cell counts for 20 control tissue sections: 16 marked with anti-CRYAB (*n* = 64 counts) and 4 marked with anti-GFAP (*n* = 16 counts), and for 29 contused tissue sections: 17 marked with anti-CRYAB (*n* = 68 counts) and 12 marked with anti-GFAP (*n* = 48 counts).

Images of immunofluorescence-labeled sections were taken with a Zeiss LSM700 Laser Scanning confocal microscope. In total, 22 counts of the proportion of cells co-expressing CRYAB and GFAP were obtained for these sections. Images for counts of astrocytes co-expressing CRYAB and GFAP were obtained with a 20X/0.5 objective lens. The number of cells expressing each marker over the full image extent was determined as well as the ratio of CRYAB/GFAP cells.

DAB-immunohistochemical images were acquired with the same filter and light intensity. Immunofluorescence images were acquired under the same conditions of laser intensity, pinhole size, signal emission, channel mode, higher resolution, scanning speed, master gain, digital gain, and digital offset. These conditions were defined by averaging the lowest and highest expression of the two markers in the contused tissue sections. The counts in the DAB-immunohistochemical and immunofluorescent markings were carried out by two independent observers (one was an expert), using the double-blind method to avoid bias and the data obtained were compared to corroborate those differences were below 5%.

### Presentation of Data and Statistical Analysis

The mean and SD of all the quantitative data were obtained for each parameter. Non-parametric Student’s *t*-tests were used to compare counts in control and TBI samples as well as for comparisons between counts of positive cells and GFAP/CRYAB. Comparisons between cortical regions were analyzed with a Kruskal–Wallis’s test and Dunn’s *post hoc* multiple comparison test. All differences were considered significant at *p* < 0.05. The measures of central tendency and dispersion, statistical analysis, and data plotting were obtained using SPSS 10.0 for Windows and GraphPad Prism 4.

## Results

### Glial Fibrillary Acidic Protein and Crystallin Alpha-B Expression in Human Contused Tissue

**FIGURE 1:**
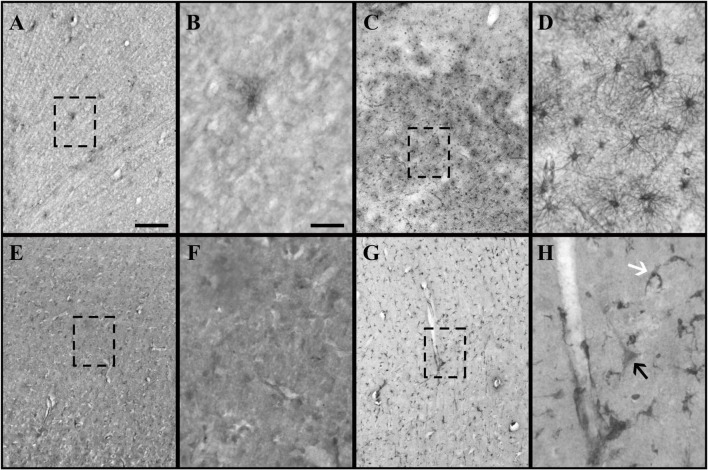
Glial fibrillary acidic protein (GFAP) and crystallin alpha-B (CRYAB) expression in the cortical gray matter of human brain tissue after traumatic brain injury (TBI). **(A–D)** GFAP expression in gray matter. **(E–H)** CRYAB expression in gray matter. **(A,B,E,F)** Control tissue. **(C,D,G,H)** TBI tissue. **(A,C,E,G)** Microphotographs with objective lens 10×/0.25, scale bar: 200 μm. **(B,D,F,H)** Enlargement of the box shown with dashed lines, with objective lens 40×/0.75, scale bar: 50 μm. The white arrow indicates a cell with astrocyte morphology. The black arrow indicates a cell with pyramidal neuron morphology.

**FIGURE 2:**
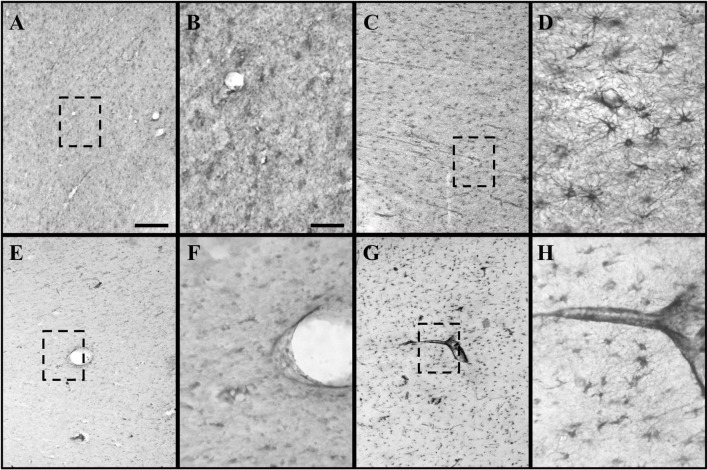
GFAP and CRYAB expression in the subcortical white matter of human brain tissue after TBI. **(A–D)** GFAP expression in white matter. **(E–H)** CRYAB expression in white matter. **(A,B,E,F)** Control tissue. **(C,D,G,H)** TBI tissue. **(A,C,E,G)** Microphotographs with objective lens 10×/0.25, scale bar: 200 μm. **(B,D,F,H)** Enlargement of the box shown with dashed lines, with objective lens 40×/0,75, scale bar: 50 μm.

**FIGURE 3:**
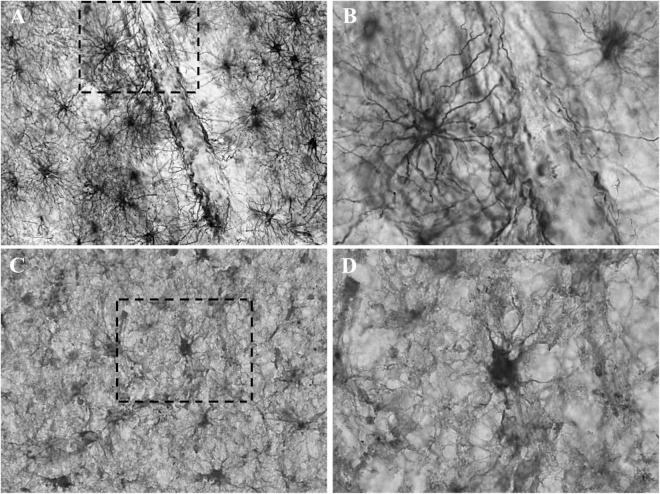
Detail of the astrocytic labeling of GFAP and CRYAB in contused brain tissue. **(A,B)** GFAP expression in the white matter of contused brain tissue. **(A)** Objective lens 40×/0.75. **(B)** Enlargement of the box shown with dashed lines, with objective lens 100×/1.3. **(C,D)** CRYAB expression in the white matter of contused brain tissue. **(C)** Objective lens 40×/0.75. **(D)** Enlargement of the box shown with dashed lines, with objective lens 100×/1.3.

Heterogeneous labeling was observed in the cerebral cortex of subjects with TBI using GFAP and CRYAB. Astrocytic forms were seen predominantly in the transition between the gray matter and the white matter, as well as in the subcortical white matter itself (see Figures 1, 2). In most cases, staining excluded the nucleus and was more intense in the cytoplasm and in the process, allowing the identification of astrocyte morphology (see Figures 2D,H, 3). The intensity of labeling was higher in TBI tissue compared with control tissue, where it was very weak or null. The processes were thickened and there was a noticeable increase in cell size (see Figures 1, 2). Both markers allowed the identification of two astrocytic forms: protoplasmic astrocytes with short and intricate processes, predominantly located in the cerebral cortex layers, and larger fibrous astrocytes with longer processes and fewer branches, located predominantly in the subcortical white matter (see Figure 3). Astrocytes associated with numerous blood vessels and end foot extensions were also visible in the subcortical white matter (see Figures 3A,B).

**FIGURE 4:**
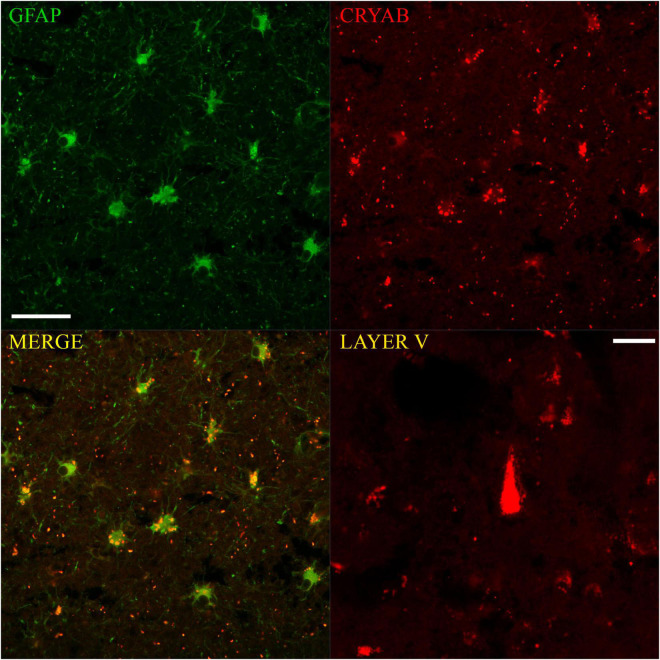
Immunofluorescent colocalization of GFAP and CRYAB expression in astrocytes of the gray-white matter transition of the cerebral cortex after human TBI. Microphotographs of CRYAB/GFAP/MERGE double labeling with 40×/1.3 objective lens and additional 1.0× zoom, scale bar: 100 μm. A CRYAB-positive cell with pyramidal morphology, located approximately in layer V of the contused cerebral cortex, is shown in the lower right box. Microphotographs with 40×/1.3 objective lens and additional 1.5× zoom, scale bar: 20 μm.

Using consecutive tissue sections marked with Anti-NeuN (the marker that allowed us to visualize the total population of cortical neurons), we identified the boundaries between cortical layers in sections marked with GFAP and CRYAB. In this way, we were able to carry out a more precise qualitative analysis of GFAP and CRYAB marking and select sectors for quantitative analysis. CRYAB-positive cells were observed on surface layers of contused cortical tissue, but they were more frequent in deep sectors of contused cortical tissue and in subcortical white matter. A few of these cells were distinguishable in layer V as neurons, due to their clearly pyramidal morphology and the orientation of their apical dendrites (see Figures 1G,H, 4). The staining of neurons was very thin. Therefore, staining was heterogeneous and always excluded the nucleus; it was seen mainly in the cytoplasm and the proximal processes of all stained cells.

### Quantitative Analysis of Glial Fibrillary Acidic Protein

**FIGURE 5:**
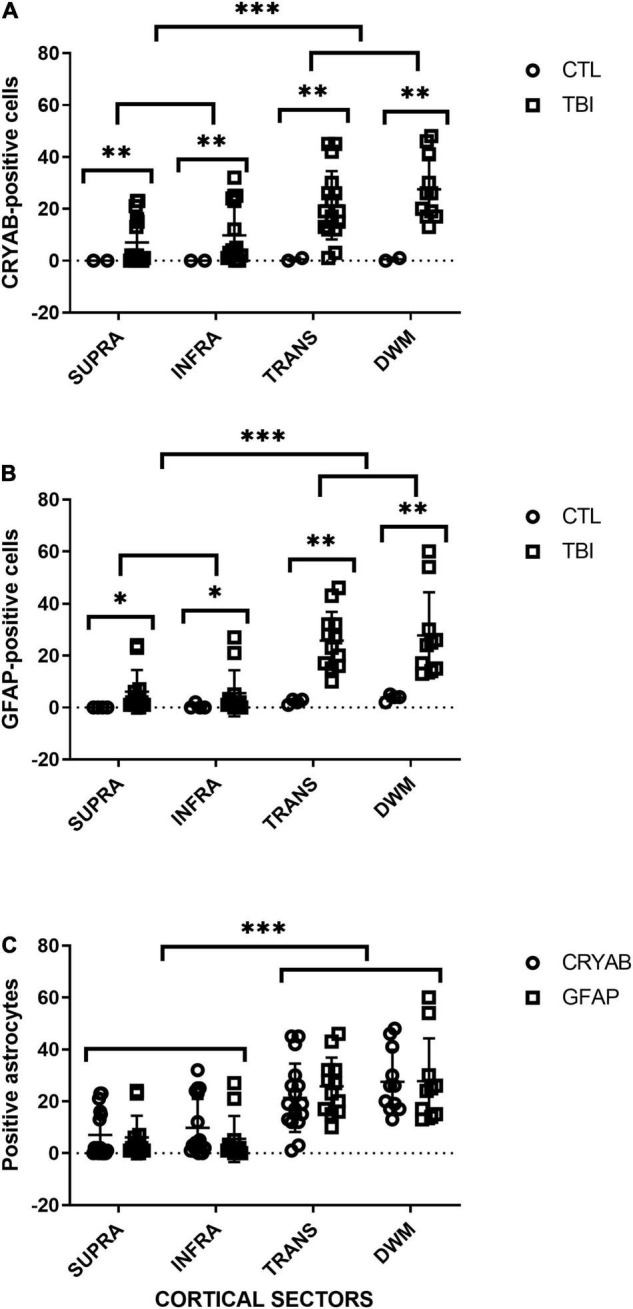
Quantitative analysis of GFAP- and CRYAB-positive cells in contused brain tissue using DAB-immunohistochemistry. **(A)** Scatterplot of CRYAB-positive cells in human postmortem and contused brain tissue. **(B)** Scatterplot of GFAP-positive cells in human postmortem and contused brain tissue. **(C)** Comparison of the number of GFAP- and CRYAB-positive astrocytes in human contused brain tissue. Cortical sectors: SUPRA (between layers II and III), INFRA (specifically in layer V), TRANS (transition between layer VI and subcortical white matter), and DWM (deep white matter). *p < 0.05, ^**^p < 0.01, and ^***^p < 0.001.

For each analyzed sector (SUPRA, INFRA, TRANS, and DWM), a significantly higher number of GFAP-positive cells were observed in the tissue of patients with trauma than in controls (SUPRA and INFRA: *p* < 0.05; TRANS and DWM: *p* < 0.01). There was little staining of control tissues, with no staining of the supragranular layers, and a progressive increase in staining in the deeper sectors of the cortex, with the highest counts in deep white matter. In injured tissues, infragranular layers had the lowest cell counts, with a very slight increase in supragranular layers, and a very large increase in white matter. There were no significant differences in the counts of the analyzed cortical sections of control tissue between individuals (*p* > 0.05). The comparison of cortical sectors of damaged tissue resulted in significant differences in cell counts between SUPRA vs. TRANS (*p* < 0.001), SUPRA vs. deep white matter (DWM) (*p* < 0.001), INFRA vs. TRANS (*p* < 0.001) and INFRA vs. DWM (*p* < 0.001). No significant differences were found between SUPRA vs. INFRA (*p* > 0.05) and TRANS vs. DWM (*p* > 0.05). The gray matter sector (SUPRA and INFRA) formed a statistical group that was clearly separate from white substance sectors (TRANS and DWM) (see Figure 5A).

### Quantitative Analysis of Crystallin Alpha-B

For each analyzed sector (SUPRA, INFRA, TRANS, and DWM) a significantly higher number of CRYAB-positive cells was observed in the injured tissue than in the controls (*p* < 0.01), with a progressive increase in the number of positive cells in deep cortical layers and white matter. The supragranular layers had the lowest number of cell counts, whereas the deep white matter had the highest number. There were no significant differences in the number of CRYAB-positive cells among sectors of the cerebral cortex in control tissue (*p* > 0.05). The comparison of cortical sectors of injured tissue resulted in significant differences in cell counts between SUPRA vs. TRANS (*p* < 0.001), SUPRA vs. DWM (*p* < 0.001), and INFRA vs. DWM (*p* < 0.001). No significant differences were found between SUPRA vs. INFRA (*p* > 0.05), INFRA vs. TRANS (*p* > 0.05), and TRANS vs. DWM (*p* > 0.05) (see Figure 5B).

### Colocalization of Glial Fibrillary Acidic Protein and Crystallin Alpha-B in Astrocytes

Confocal microscopy allowed the observation of double labeling of CRYAB and GFAP proteins at the cellular and subcellular level in tissues of subjects who suffered TBI. There were clear differences in staining: GFAP labeling was almost always of the cytoplasmic content in all its apparent extension and allowed the observation of the proximal and distal processes, whereas CRYAB labeling allowed, in most cases, the identification of intracytoplasmic inclusions in a dotted manner. On the other hand, fluorescence also showed very few cells with pyramidal morphology positively marked for CRYAB in layer V of the cortex, which corroborates the data obtained with DAB-immunohistochemistry (see Figure 4).

The comparison of the GFAP- and CRYAB-positive cell counts for each cortical region of injured tissue showed no significant differences between immune profiles for the two markers (*p* > 0.05) (see Figure 5C). We did not quantify the proportion of cells with double and single labeling because we did not have a similar number of sections labeled with immunofluorescence and with DAB-immunohistochemistry. However, we were able to qualitatively observe that the double-labeled cells varied according to the sector of the section analyzed, with colocalization being minimal in the SUPRA sectors and maximal in the TRANS sectors. In the latter, between 35 and 100% of cells colocalized for both markers, and all showed astrocytic morphology (see Figure 4).

Crystallin alpha-B fluorescent labeling showed many “extracellular” red dots (see Figure 4), which was the predominant form of labeling in the SUPRA and INFRA sectors of some of the sections. Some of these red dots corresponded to the somatic labeling of cells located on different focal planes from the microphotographs; other points were clearly located outside the astrocytes. The latter could be located in β-amyloid deposits (Aβ-40 and Aβ-42), which were abundant in sections of contused tissue samples (unpublished data) and have also been reported in Alzheimer’s disease (Narayanan et al., 2006).

## Discussion

### Cellular Features Found With Crystallin Alpha-B Immunolabeling: Reactive Astroglia

The similarity in counts of GFAP- and CRYAB-positive cells, as well as the cellular colocalization of both markers found in this study, allow us to propose CRYAB as a possible alternative histological biomarker of reactive astrogliosis in TBI.

Reactive astroglia are characterized by increased cell size and the production of cytoskeletal intermediate filaments; for this reason, GFAP labeling has been widely used to identify this process in a variety of central nervous system disorders, including TBI (Sofroniew, 2009; Sofroniew and Vinters, 2010; Burda and Sofroniew, 2014; Pekny and Pekna, 2014; Liddelow and Barres, 2017). Axotomy as a consequence of trauma is apparently one of the triggers of reactive astrogliosis even in areas distant from the site of injury (Steward and Trimmer, 1997). In this study, GFAP labeling showed that tissue sections from different subjects could be interpreted as mild to moderate astrogliosis in some cases, or severe diffuse astrogliosis in others, according to the classification proposed by Sofroniew and Vinters (2010). None of the samples from subjects who suffered trauma showed severe reactive astrogliosis characteristics with glial scar formation that could be due to trauma evolution, if glial scars were to be considered a phenomenon that can occur over time.

In this study, the cell morphology and distribution of CRYAB-positive cells were qualitatively similar to those reported in other GFAP studies. No significant differences were found between counts of supragranular and infragranular regions with antibodies against CRYAB and GFAP. This may indicate that the astroglial reactivity process tends to be homogenous in the injured section, unlike the selective vulnerability of neurons according to the cortical layer seen in TBI (Riascos et al., 2013). It should be noted that for both GFAP and CRYAB labeling, expression was always higher in subcortical white matter than in cortical gray matter, possibly because the number of astrocytes was higher in subcortical white matter. Axotomy is an integral part of the pathophysiology of TBI, and the astroglial reactivity observed in the white matter might be due to axonal injury of neurons with cell bodies in layers III or V, or to damage to cortical afferents. We can conclude that GFAP and CRYAB labeling does not correlate with the degree of the neuronal lesion between layers of the same cortical sector, but general labeling of both gray and white matter may be physiologically related to damage to the entire sector.

### Expression of Glial Fibrillary Acidic Protein and Crystallin Alpha-B in During the Subacute Stage of Traumatic Brain Injury

Traumatic brain injury samples used in this study were obtained from patients 48 to 72 h posttrauma (Table 1). Several studies using animal models have established that after direct tissue damage caused by trauma, a complex progression of the injury develops, known as secondary damage, which presents different characteristics depending on the post-trauma time period (Cernak et al., 2004; Hellewell et al., 2010). Hypotension and hypoxia are present during secondary damage; they are responsible for the increase in noxa. It has been reported that neuronal responses directly due to injury tend to be more intense during the first few hours after trauma. However, at later stages, beginning approximately 12 h after injury, there is increased astroglial involvement and its cellular response to injury; this has been shown by using GFAP as a marker. It is important to clarify that some studies have shown that GFAP is not an exclusive marker of astrocytes, although it is a marker of most astrocytes. The definitive categorization of a given cell as an astrocyte could be achieved through the use of joint markers, as is the case when using GFAP staining with AQP4 (Ikeshima-Kataoka, 2016; Kitchen et al., 2020). Astroglial involvement has also been observed in ischemia (Piao et al., 2005; Li et al., 2012), where early CRYAB neuronal immunolabeling in the cerebral cortex of the rats and CRYAB astroglial labeling in the cortex and subcortical white matter beginning 24 h postinjury were identified.

Glia plays a key role 24 h postinjury in the pathophysiology of trauma and ischemia; their biological activity increases to restore environmental conditions. During secondary damage, the glial network could favor the buffering of ions and glutamate uptake, distributing substances through the network. If this phenomenon persists, the connections system could also behave as another aggravating factor in the process, since the substances that are transported between connections, such as glutamate and calcium ions, among others, could induce death cascades in glia and the release of proinflammatory or toxic substances in sectors that may be focal or exofocal, contributing to the spread of damage (Burda et al., 2016). In our group, a study of cerebral ischemia using animal models showed a significant decrease in the DAB-immunohistochemical expression of glial glutamate transporter GLT1 in layer III of the cerebral cortex contralateral to the ischemic core after occlusion of the middle cerebral artery, which could contribute to the hyperexcitability of sectors that are distant to the ischemic core (Medina et al., 2008). These changes were observed just 24 h postinjury and increased up to 72 h after ischemia, corresponding to the same period analyzed for TBI in this study. These changes are probably due to the deafferentation of commissural fibers that originate in sectors of ischemic focus and that can be correlated with axotomy in TBI.

### Crystallin Alpha-B as a Stabilizer of Intermediate Filaments

Double labeling of CRYAB and GFAP proteins in contused tissue using confocal microscopy showed the coexistent overexpression of both markers. This co-expression of the two proteins has also been demonstrated for Rosenthal fibers in Alexander’s disease, chronic glial scars, low- and high-grade fibrillary astrocytoma, etc. (Der et al., 2006; Hagemann et al., 2006; Goplen et al., 2010). Likewise, there are reports of GFAP overexpression in mice, indicating that the accumulation of intermediate filaments upregulates the expression of sHSPs. It has been shown that CRYAB controls the monomer–monomer interactions established between the intermediate filament proteins to prevent their pathological aggregation (Der et al., 2006). This would explain the increased expression of both proteins in trauma and cellular co-localization found in this study, as an attempt of CRYAB to stabilize the characteristic increase in intermediate filaments during the gliosis process to prevent its collapse.

However, other studies have found different functions and cytoplasmic locations of CRYAB. For example, CRYAB has been found to inhibit autoproteolytic maturation of caspase 3; it colocalizes *in vivo* with the intermediate product of this catalysis, peptide p24 (Kamradt et al., 2001; Shin et al., 2009). On the other hand, some data have indicated that CRYAB accompanies the disassembly of the Golgi apparatus during prometaphase, and its reassembly once cytokinesis is complete, as well as its presence in this membrane complex during interphase (Gangalum et al., 2004). Its function during interphase in this organelle is probably related to its role as a chaperone, considering the activity of the Golgi apparatus as the main site of post-translational processing of proteins and as a targeting station for secreted proteins. den Engelsman et al. (2003) described the interaction of CRYAB with FBX4, a component protein of the SKP1/CUL1/F-box (SCF) ubiquitin ligase protein (SKP1/CUL1/F-box); that is, a protein that is responsible for capturing and signaling target proteins so they undergo a degradation process through the proteasomal pathway (den Engelsman et al., 2003). This interaction is strengthened when CRYAB is phosphorylated at serine 19 and 45, which occurs mainly during the mitotic phase of the cell cycle; it would, therefore, be of greater importance in astrocytes than in neurons in the nervous system.

### Crystallin Alpha-B Expression in Neurons During the Post-trauma Subacute Period: Selective Vulnerability

Few CRYAB-positive cells with pyramidal morphology were observed in layer V of the cerebral cortex of injured samples in this study. Layer V of the cerebral cortex contains large pyramidal neurons that project to other cortical regions and to distant areas of the system, such as the striatal nucleus, brainstem nuclei, and spinal cord. Escobar et al. (2008) found that layers III and V of the cerebral cortex were more likely to show alterations of the microvasculature in human TBI. They also reported lower cell counts and greater loss of IR quality when using NeuN and MAP2 labeling in layer V than in other cortical layers. Pyramidal neurons may be compromised by axotomy because of the kinetics of trauma. The vulnerability of the inner pyramidal layer could induce the expression of proteins related to cell survival as part of the response to injury, which would explain the presence of CRYAB IR in the injured tissue. The only difference between the quantitative CRYAB and GFAP profiles found in this study was that there were no significant differences in cell counts between the infragranular layers of the cortex (INFRA) and the transition between layer VI and white matter (TRANS) for CRYAB; and on the contrary, there were significant differences between these two cortical regions for GFAP. This small difference could be due to the number of CRYAB-positive neuronal cells found in the infragranular layers of the cortex.

Although most studies have indicated that many diseases cause CRYAB overexpression in astrocytes, studies of cerebral ischemia in murine models (Piao et al., 2005; Li et al., 2012) showed an early expression of CRYAB in pyramidal neurons of the cerebral cortex during the first hours after occlusion of the middle cerebral artery and reperfusion. Approximately 24 h after occlusion, there was the induction of CRYAB expression in reactive astrocytes, which was maintained for several days. These findings allow us to suggest that CRYAB expression could occur in neuronal cells during early TBI, considering the relationship between CRYAB and ischemic events at the pathophysiological level. If so, the regulatory mechanisms of expression or the activation of the protein may be different in glia and neurons. However, the data presented in this work correspond to the subacute period of trauma, since contusion extraction was carried out between 48 and 72 h after injury, according to medical criteria for each case, which generally coincides with contusion growth and/or bleeding peaks.

## Implications/Outlooks

No statistically significant differences were found in cell counts of cortical sectors between cells that showed CRYAB and GFAP expression in this study, using DAB-immunohistochemical methods. In addition, we corroborated the astrocytic colocalization of the two proteins (CRYAB and GFAP) through immunofluorescent double-labeling observed with confocal microscopy. The described characteristics of CRYAB labeling allow us to propose it as a possible tissue indicator of reactive astrogliosis during the subacute period of human TBI.

Although this study focused on the evaluation of CRYAB as a possible marker of reactive astrogliosis in cerebral contusions, this protein has other specific functions evaluated in other contexts or pathologies (as mentioned above), which could be evaluated by markers of apoptotic or inflammatory events in the same tissue. The possible subcellular colocalization of the CRYAB protein in tissue from trauma subjects could be assessed using immunofluorescence or immunoprecipitation with markers of these events, contributing thus to a more contextual assessment of the role of this protein in human TBI.

This study evaluated changes in the protein expression of CRYAB during the subacute period of TBI, as patients underwent surgery between 24 and 72 h postinjury, according to medical criteria and evolution. The evaluation of the expression of this protein at other stages (before or after the stage evaluated here) could be undertaken using non-human animal models of TBI. This, in turn, would allow an in-depth discussion of some of the approaches mentioned above, including the possible early expression of CRYAB in other non-astrocytic nerve cell types. Likewise, the overexpression of CRYAB as a possible response to GFAP upregulation could be verified using GFAP knock-out mice.

## Data Availability Statement

The raw data supporting the conclusions of this article will be made available by the authors, without undue reservation.

## Ethics Statement

The study involving human participants was reviewed and approved by the Human Ethics Committee of the Faculty of Health, Universidad del Valle, Cali, Colombia. Written informed consent to participate in this study was provided by the participants’ legal guardian/next of kin.

## Author Contributions

LVB-H, MIE-B, and HJP-J had the idea for the article. LVB-H and HJP-J carried out the literature search. LVB-H and EB carried out the experiments. LVB-H, EB, MIE-B, and HJP-J analyzed the data. LVB-H wrote the first draft of the manuscript. EB, MIE-B, and HJP-J critically reviewed this study. All authors contributed to the article and approved the submitted version.

## Conflict of Interest

The authors declare that the research was conducted in the absence of any commercial or financial relationships that could be construed as a potential conflict of interest.

## Publisher’s Note

All claims expressed in this article are solely those of the authors and do not necessarily represent those of their affiliated organizations, or those of the publisher, the editors and the reviewers. Any product that may be evaluated in this article, or claim that may be made by its manufacturer, is not guaranteed or endorsed by the publisher.

## Supplementary Material

Supplementary Figure 1 Validation of crystallin alpha-B (CRYAB)-gene overexpression through quantitative PCR. ^###^, ^+++^, ****p* < 0.001. ^###^Statistically significant difference between traumatic brain injury (TBI) temporal cortex samples and TBI frontal cortex samples. ^+++^Statistically significant difference between TBI temporal cortex samples and control frontal cortex samples. ***Statistically significant difference between TBI temporal cortex samples and control temporal cortex samples.
