# Supplementary figures and images for: Crystallin Alpha-B Overexpression as a Possible Marker of Reactive Astrogliosis in Human Cerebral Contusions
Source: Front Cell Neurosci. 2022 Mar 14;16:838551. doi: 10.3389/fncel.2022.838551 (PMC8963874; doi:10.3389/fncel.2022.838551)

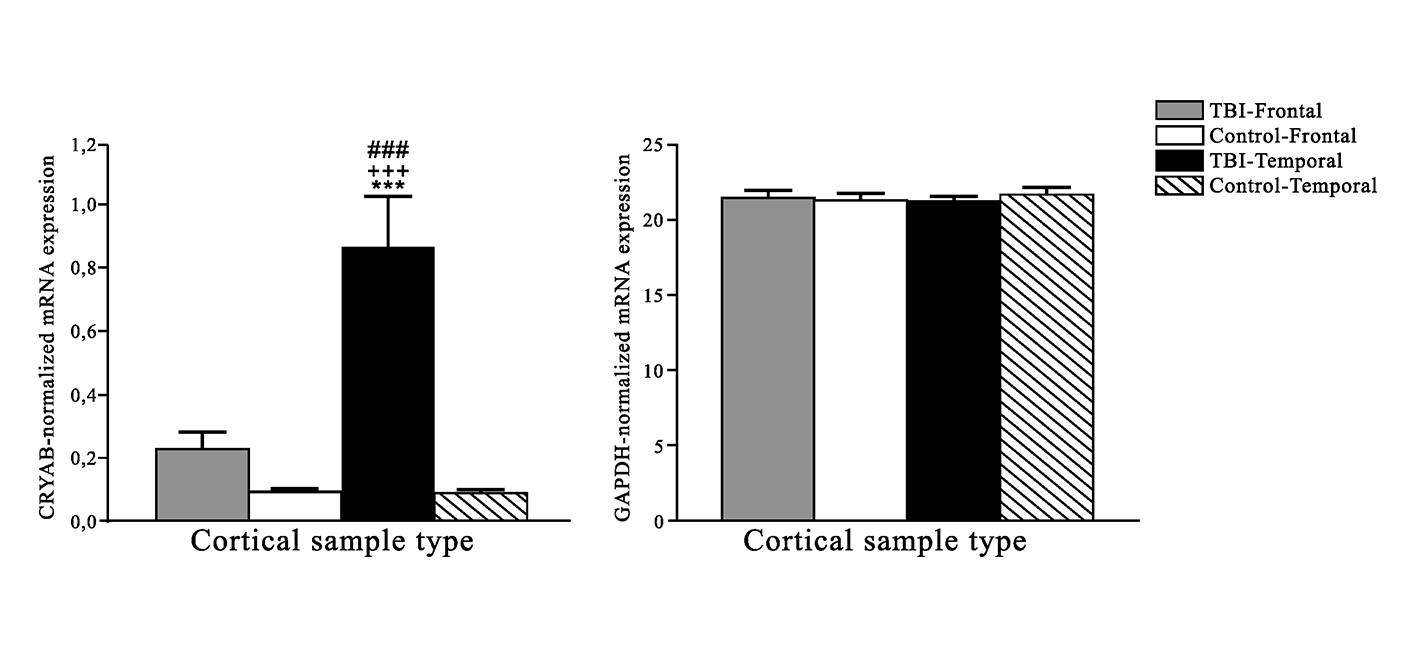

Supplement: Supplementary Figure 1 — Validation of crystallin alpha-B (CRYAB)-gene overexpression through quantitative PCR. ###, +++, ***p < 0.001. ###Statistically significant difference between traumatic brain injury (TBI) temporal cortex samples and TBI frontal cortex samples. +++Statistically significant difference between TBI temporal cortex samples and control frontal cortex samples. ***Statistically significant difference between TBI temporal cortex samples and control temporal cortex samples. [file Image_1.TIF]
